# Omega 3 Fatty Acids and Health: The Little We Know after All These Years

**DOI:** 10.3390/nu14020239

**Published:** 2022-01-06

**Authors:** Francesco Visioli, Carlo Agostoni

**Affiliations:** 1 Department of Molecular Medicine, University of Padova, 35122 Padova, Italy; francesco.visioli@unipd.it; 2 IMDEA-Food, 28049 Madrid, Spain; 3 Pediatric Area, Fondazione IRCCS Ca’ Granda—Ospedale Maggiore Policlinico, 20122 Milan, Italy; 4 Department of Clinical Sciences and Community Health, University of Milan, 20122 Milan, Italy

Elagizi et al. [1] provide an update on the effects of long-chain omega 3 fatty acids on the cardiovascular system. In a way, the need for an update is a bit surprising given that omega 3 research dates back to the early 1970s [2] and that the omega 3 fatty acids docosahexaenoic (DHA) and eicosapentaenoic (EPA) are among the most studied substances [3]. However, as Elagizi et al. correctly conclude, there is no consensus on their role in cardioprotection and in oncology [1]. The big question is why? What went wrong after the initial reports, e.g., GISSI-Prevenzione [4] or JELIS [5], of the positive actions of omega 3 fatty acids in patients with cardiovascular infarction (MI)? We propose some working hypotheses that should be addressed in future investigations.

One is that fatty acid concentrations are almost never measured before and after trials [6]. From a mere pharmacological point of view, this is surprising because, for example, the HS-Omega-3 Index^®^ (%EPA and DHA in erythrocytes, measured with a standardized methodology) is a risk predictor of cardiovascular mortality (CVD) [7], possibly stronger than total cholesterol [7]. Namely, CVD is 30% lower with an Omega-3 Index >8% as compared with an Omega-3 Index of <4% [8]. In the original trials, omega 3 capsules might have been given to people who did not need them and too low of a dose might have been administered to people with a very low Omega-3 Index. This resembles running hypertension trials without measuring blood pressure or hyperlipidemia trials without measuring low-density lipoproteins (LDL).

Along these lines, most if not all authors neglect the issue of bioavailability. As reviewed by Schuchardt and Hahn [9], bioavailability heavily depends on the concomitant intake of fat and/or adequate volume of foods, has high interindividual variability, etc. Examples of good bioavailability include omega-3 fatty acids formulated in milk products [10] or when eaten with salmon [11] versus capsules, both not used in the meta-analyzed trials. Interestingly, epidemiological studies agree that fatty fish consumption is moderately but inversely correlated with CVD [12].

Another issue is the concomitant pharmacological therapy. When the GISSI-Prevenzione study was conducted, only 4% of patients received statins (following the 4S trial) and probably fewer than those with a stent implanted. Nowadays, patients with MI are polytreated with strong drugs, masking any potential effect of fish oil [6].

Another topic to which we would like to introduce the reader (and elegantly addressed by Elagizi et al. [1]) is that of the differential effects of EPA and DHA. Although usually administered together, recent trials with high-dose pure EPA reported cardioprotection [13] so that, in November 2019 [14], the FDA approved the use of pure EPA in patients with hypertriglyceridemia [15], expanding the ‘the lower, the better’ cholesterol guideline [16]. Maybe future trials and basic investigations should discriminate the effects of these two fatty acids, which were forerun by the JELIS study [5].

The mechanisms of action of EPA and DHA are also far from being elucidated. They definitely reduce triglyceride concentrations and are very useful in familiar hypertriglyceridemia. They also have anti-inflammatory actions, which could be shared by alpha-linolenic acid (ALA) [17], although studies on this fatty acid are suggestive but scarce [18]. As inflammation is one of the main contributors to degenerative diseases, frequent intake of EPA and DHA could contribute to a better prognosis [19]. Unfortunately, once CVD is established, it becomes difficult to see the strong therapeutic effects of fish oil administration. Last, but not least, EPA and DHA have been credited with anti-arrhythmic activities, as shown by in vitro studies [20] and early human trials [20]. However, recent evidence shows the exact opposite, i.e., fish oil administration increased atrial fibrillation in a meta-analysis of secondary prevention RCTs [21]. This effect was stronger in high-risk patients and in those with elevated plasma triglycerides [21].

In conclusion, maintaining appropriate essential fatty acid, i.e., omega 3 [12,19] and omega 6 [22], intake affords better cardiovascular health. However, the enthusiasm that accompanied the first RCTs of EPA and DHA is fading, as the use of pharmaceutical preparations in secondary prevention did not lead to clear preventive effects [23]. Future research should concomitantly focus on mechanisms of action and on finding whether there is a difference between EPA and DHA (and, in the future, ALA) in terms of cardioprotective effectiveness. Finally, we strongly advocate measurements of omega 3 concentrations, e.g., the Omega-3 Index, to discriminate among CVD patients and before solid conclusions on the effectiveness of omega 3 fatty acids in CVD therapy are drawn.
